# What is in the toolkit (and what are the tools)? How to approach the study of doctor–patient communication

**DOI:** 10.1136/postgradmedj-2021-140663

**Published:** 2022-01-05

**Authors:** Caitríona Cox, Zoë Fritz

**Affiliations:** The Healthcare Improvement Studies Institute, University of Cambridge, Cambridge, UK; The Healthcare Improvement Studies Institute, University of Cambridge, Cambridge, UK

**Keywords:** statistics & research methods, qualitative research, medical education & training

## Abstract

Doctor–patient communication is important, but is challenging to study, in part because it is multifaceted. Communication can be considered in terms of both the aspects of the communication itself, and its measurable effects. These effects are themselves varied: they can be proximal or distal, and can focus on subjective measures (how patients feel about communication), or objective measures (exploring more concrete health outcomes or behaviours). The wide range of methodologies available has resulted in a heterogeneous literature which can be difficult to compare and analyse. Here, we provide a conceptual approach to studying doctor–patient communication, examining both variables which can controlled and different outcomes which can be measured. We present methodologies which can be used (questionnaires, semistructured interviews, vignette studies, simulated patient studies and observations of real interactions), with particular emphasis on their respective logistical advantages/disadvantages and scientific merits/limitations. To study doctor–patient communication more effectively, two or more different study designs could be used in combination. We have provided a concise and practically relevant review of the methodologies available to study doctor–patient communication to give researchers an objective view of the toolkit available to them: both to understand current research, and to conduct robust and relevant studies in the future.

## Introduction

Despite communication being integral to the doctor–patient relationship, it has not always been subject to the rigorous scientific evaluation applied to other aspects of medicine [1]. Research has demonstrated correlative links between good communication and a range of effects (including improved patient satisfaction, trust and health outcomes) [2–4], but thorny questions remain: what constitutes ‘good communication’ in a global sense? What changes do patients want to see in practice? How can the impacts of different communication behaviours be assessed in different clinical settings?

Doctor–patient communication is challenging to study, or even to define as a construct: it is multifaceted, consisting of variables relating to both style and content. Outcome measures are similarly varied: some studies focus on subjective measures (patient responses to communication), while others use objective measures (exploring the impact of communication on more concrete health outcomes or behaviours) [3, 5]. Different methodological approaches have resulted in variable quality of evidence, and difficulties in directly comparing results.

A number of papers have provided detailed analysis of specific instruments or study designs used to examine doctor–patient communication [6–8]. Here, we provide a general overview including (1) a conceptual approach to the study of doctor–patient communication and (2) a summary of some of the tools and study designs which can be employed. We analyse the logistical advantages/disadvantages and scientific merits/limitations of different approaches, providing researchers with an objective view of the toolkit available to them.

## What aspects of communication can be studied?

**Figure 1:**
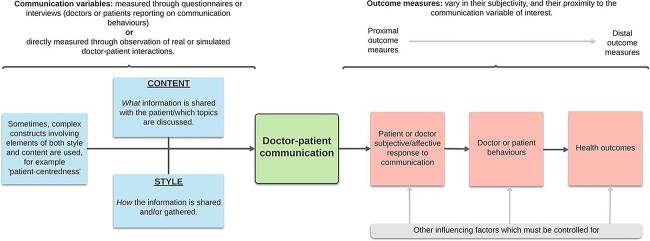
Communication variables and outcome measures: a schematic diagram depicting how different methodologies need to be adopted to assess content, style and outcomes of communication.

Communication can be considered in terms of variables (aspects of communication style and content which can vary), and outcomes (measurable effects of communication). It is important to determine which aspects are being investigated in order to choose the most suitable methodologies. Figure 1 is a schematic diagram depicting how different methodologies need to be adopted to assess content, style and outcomes of communication.

### Communication variables

As Street *et al* note, ‘(at) the most basic level, different operational and conceptual definitions of the variables to be measured are problematic’ [9]. Communication can be broadly considered in terms of content (what information is given) and style (how information is given), although viewing the two as totally separate can create a false dichotomy: patients are influenced by both in a way which can be difficult to separate [3]. 

The content of communication can be manipulated experimentally or measured in observational studies, for example, by assessing whether a specific topic is discussed. This is more complex if poorly defined constructs are used (eg, measuring whether ‘diagnostic uncertainty’ is communicated). Instruments for measuring the content of communication are often checklist-based (is topic × communicated?), and can involve quantitative measurement (how often is topic × mentioned?). For example, researchers have analysed transcriptions of consultations to measure how often prognostic information was provided [10]. 

The style of communication is more difficult to manipulate experimentally or measure in observational studies, partly because it is more difficult to define. Verbal and non-verbal elements must be considered. The occurrence and frequency of specific behaviours which contribute to the overall style can be measured: a behavioural-coding approach can be used, categorising units of speech and recording how often they occur. An example of this is a study which analysed audiotaped consultations using the Roter interaction analysis system patient-centredness subscale [11]. The consultation was divided into units of speech; each unit was given three codes, to characterise source (doctor, patient or third party), process (open/closed questions, initiated statements and question responses) and content (diagnosis, prognosis, treatment, medical history and presenting symptoms, other medical matters, social matters and other). The ratio of doctor to patient speech was compared, among other analyses.

More commonly, rating scales are used to provide more global assessments of communication style. Evaluators—who could be external observers, or the patients themselves—provide overall ratings on a Likert scale. For example, in a study using rating scales physician-patient interactions were observed, and were scored using a 4-point scale on a range of verbal and non-verbal behaviours including empathy, physical attention and listening [12]. Compared with behaviour-coding methods, rating scales are often less time-consuming and provide a more holistic assessment, but are more subjective [5, 6, 13]. Moreover, they often do not provide information on what specific elements of the communication behaviour contributed to the overall evaluation [13]. 

Complex constructs—consisting of elements relating to both style and content—can be used. Perhaps the most widely studied example is ‘patient-centredness’ [5, 14–18]. This has been used to describe communication which affords greater importance to patient perspectives and permits patient involvement in interactions [15]. A large number of instruments have been developed to measure it experimentally [5, 16]. There are, however, significant differences between the various scales, and conceptual inconsistencies in definitions of patient-centredness have resulted in tools which—despite a common label—differ in exactly what is assessed, and how it is assessed [13]. ‘Patient-centredness’ has thus been criticised as lacking in a ‘unified definition and operationalised means of measurement’ [16]. 

A final point to consider regarding communication variables is the importance of considering the ontological assumptions underlying measurement [13]. For example, in using a behavioural-coding approach, you assume that communication is made up of discrete acts which can be measured by observers; an evaluative recall measure implies that communication behaviours exist as perceptions which can be recalled and interpreted.

#### Outcomes

Many researchers are not only interested in measuring the different communication variables, but also their effects, by examining associations between certain aspects of communication and potential consequences [4, 19–22]. Outcome measures can be proximal or distal, depending on the temporal relationship to the communication variable of interest (see Figure 1).

Proximal outcome measures tend to be subjective and are typically measured using questionnaires or interviews. They often focus on affective responses to communication behaviours. Overall patient satisfaction is the most recognised and widely used [3, 23]; other proximal outcomes which have been studied include patient trust [24] and patient perception of doctor competence [24]. 

Intermediate outcomes are those concerned with doctor or patient behaviours (such as treatment concordance or investigation/referral rates) [20, 25]. These can be measured subjectively (eg, asking a patient to report on treatment compliance using a questionnaire or an interview), or objectively (eg, by measuring how often a prescription is filled).

Distal objective outcome measures examine health outcomes; examples can be seen in box 1 [4, 21]. Both subjective and objective measures of distal health outcomes are of importance: ‘outcome measures should include both objective measures of disease process and self-report of illness experience where appropriate’ [26]. Overall, examining health outcomes can be useful in providing evidence about associations between certain communication behaviours and improved health. These studies must be carefully controlled, as many factors impact on distal health outcomes and confounding factors may have a significant effect [9].

Box 1 Outcome measures which have been used in studies of doctor–patient communication:

1. Subjective measures:
  - Patient overall satisfaction [23].
  - Patient decisional conflict [55].
  - Patient perception of doctor competence [24].
  - Patient trust in their doctor [24].
  - Patient anxiety [56].
  - Patient perception of symptom burden/resolution [57].
  - Physician evaluation of symptom burden/symptom resolution [58].
2. Objective measures:
  - Investigations ordered (eg, chest X-ray ordered after consultation) [59].
  - Medication prescribed (eg, antibiotics prescribed per consultation) [59].
  - Patient recall of information/knowledge [55].
  - Laboratory/physical measurements (eg, blood pressure [60], haemoglobin A1c) [61].
  - Patient functional impairment/physical limitation [62].
  - Organ damage (eg, Systemic Lupus International Collaborating Clinics/American College of Rheumatology Damage Index measure of cumulative organ damage in SLE (systemic lupus erythematosus). [22]
  - Length of intensive care unit (ICU)stay [63].
  - Referral/reattendance rates [20].
  - Concordance with treatment [25].

Whether the focus of communication studies should be on proximal or distal outcomes is debatable. Many have emphasised the importance of promoting patient-centredness in doctor–patient communication [5, 15]. Some view it as a means to an end: without changes in intermediate or distal outcomes, they argue that changes in communication behaviour are not important in themselves. In contrast, others maintain that patient-centredness is an end-in-itself, with intrinsic value regardless of other outcomes [15]. According to this conceptualisation, proximal outcomes alone are of sufficient value to study.

## Specific examples of data collection tools and study designs

A range of study instruments and designs have been utilised in research examining doctor–patient communication. Some of the logistical considerations, and scientific merits and limitations, of different methodologies are outlined in Table 1; we further discuss these below with illustrative examples.

**Table 1:** An overview of the advantages and disadvantages of available methodologies for the study of doctor–patient communication

Method	Logistical considerations	Scientific merits	Scientific limitations
Questionnaires	Relatively inexpensive and quick to administer. Can suffer from low-response rates.	Flexible: can be designed to assess many different aspects of communication, and can be administered to doctors or patients. Analysis can be quantitative or qualitative, depending on the questionnaire design.	Questionnaires alone only provide information on reported behaviours: they do not allow for direct observation or measurement of communication behaviours. Although they can have open questions, questionnaires tend not to produce as rich or detailed data as other qualitative methods, such as interviews. There is wide variation in how questionnaires are developed and validated: a range of different questionnaires have been used to assess various aspects of communication, which are of variable quality. Questionnaires can be susceptible to various biases (eg, social desirability bias).
Interviews	More time-intensive than questionnaires and require trained interviewers. Generally less expensive than observational, simulated patient or vignette studies.	As with questionnaires, interviews are flexible can be used to assess a no of different measures. They allow more detailed exploration of themes compared with questionnaires.	Interviews alone can again only provide information on reported behaviours or experiences. There can be inconsistency between interviews with different participants; different interviewers may systematically elicit different responses from participants. Difficulties arise in avoiding potential interviewer and coder biases, and in establishing reliability and validity. In focus groups, group dynamics can influence data collection.
Vignette Studies	Can be quicker and less costly than observational studies. Can still incur significant costs, in particular when actors are used in video vignettes.	Vignette studies permit greater control and standardisation of communication variables compared with real consultations: they allow experimental manipulation of specific aspects of communication. They provide an ethically acceptable method for studying potentially harmful communication behaviours. The use of analogue patients can overcome ceiling effects.	Attempts to isolate specific aspects of communication can result in an oversimplification of complex real-life doctor–patient communications. Internal validity and external validity must be established. Vignette studies cannot capture the influence of the long-term doctor–patient relationship on communication. They cannot be used to study the impact of communication on actual health outcomes.
Simulated Patient Studies	Quicker and less expensive to run than observational studies. Require trained actors, and often need significant input from both experts and lay people to develop realistic simulated patient responses.	Simulated patient studies allow for the direct study of real healthcare professionals communicating in certain controlled situations. There is greater experimental control over patient variables compared with observational studies of real patients. They are relatively flexible, and scripts can be adapted to be specific to the specialty of interest. Unannounced simulated patients can be used to increase the external validity of the study by making the simulated consultation as realistic as possible. There can be assessment of both verbal and non-verbal behaviours.	As with vignette studies, there can be concerns over both internal and external validity. These studies can only give information on how doctors communicate in response to the simulated patients—they cannot be used to study the impact of communication on actual patients or on health outcomes. As with vignette studies, the influence of the long-term doctor–patient relationship on how communication occurs and is perceived cannot be studied.
Direct observation of real consultations	Can be time-consuming to run. Involve real patient–doctor interactions, so require additional levels of ethical approval compared with simulated patient or vignette studies.	These studies involve real patients and doctors, so reflect actual clinical practice to a greater extent than simulated patient or vignettes studies. They can be tailored to examine communication in particular settings of interest (eg, communication in primary care, outpatient clinic). They permit analysis of consultations, and examination of associations between communication variables and both objective and subjective outcome measures. The use of real patients and doctors can allow for examination of communication in the context of real doctor–patient relationships: the interaction between the length of the relationship and communication can be studied, in contrast to experimental techniques such as vignette or standardised patient studies.	There may be systematic differences between patients/doctors who consent to partaking in these studies and those who do not. The Hawthorne effect must be considered. Often, these studies do not allow for controlled manipulation of relevant variables, so are observational rather than interventional. They can provide information about correlations between physician and patient communication behaviours and different outcomes measures, but do not necessarily provide any information about causation. There is a need to carefully control for confounding factors (such as patient’s baseline health). As with simulated patient studies, there is wide variation in how communication behaviours in the observed consultation are measured and analysed.

### Instruments for gathering data on patient or doctor perspectives

Perhaps the most straightforward approach to studying communication is to ask doctors, or patients, about their perspectives and experiences. Questionnaires and interviews are not only of use in isolation: for example, vignette studies use them to gather data on participant views about the provided vignettes, while studies involving observation and analysis of real consultations often also provide questionnaires to participants after the consultation.

In some situations, however, these methods of data collection are used alone, without any associated direct observation or analysis of a communication interaction. In such studies, participants can be asked to consider a recent specific communication interaction, or they can be asked to respond more generally. A problem with using either interviews or questionnaires alone is that they can only provide information on *reported* behaviours or experiences: they provide data on how patients or doctors perceive communication, without direct observation or measurement of communication behaviours. For example, in a study using interviews to explore patient experiences of cancer diagnosis, patients reported a period of ‘traumatic uncertainty’ in which the reason for investigations was not explained to them—yet without any observation of the consultations in question, it is impossible to know whether this information was simply not provided, or it was provided in such a way that patients did not understand or process it [27]. 

#### Questionnaires

Questionnaires are flexible and can be designed to assess various aspects of doctor–patient communication. They can used after interactions, asking doctors/patients to rate different elements of communication based on their experiences of the encounter; they can also be administered prior to interactions, to assess desires or preferences for communication. Paired-questionnaires, given to both doctors and patients, can be used to measure concordance between doctors' and patients' views, providing a proxy measure for communication [28]. 

Questionnaires administered to patients are particularly useful in providing information about patient perceptions of communication. They can be context-specific: Engelberg *et al*, for example, designed a Quality of Communication questionnaire, developed specifically to assess patient perceptions of communication at the end of life [29]. 

Questionnaires must be validated, and consideration given to factors including internal consistency (test-retest reliability can examine the consistency of responses, providing an indication of stability over time) [30, 31], and face or construct validity (which can be assessed using the multitrait-multimethod matrix, which examines convergence and discriminability) [32]. A number of reviews have highlighted the variable quality of questionnaires used to assess communication [14, 33]. A checklist has been developed to evaluate the quality of health measurement instruments, such as questionnaires designed to assess doctor–patient communication [34]. 

Questionnaires can suffer from low-response rates; careful thought must be given to how, and to whom, they will be distributed. They are susceptible to biases including response bias, social desirability bias, acquiescence bias and question-order bias [35–37]. Social desirability bias, for example, may result in doctors may self-reporting their own communication behaviours as better than they are in reality: ‘As the characteristics of good interpersonal care are increasingly defined and disseminated by professional and patient groups… social desirability may mask real differences between doctors by encouraging particular responses from all doctors’ [5]. 

#### Interviews

Interviews involve directly questioning patient/doctors, and can be designed to explore communication preferences or experiences. In a study examining the communication of cancer diagnosis, for example, patients were interviewed within 1 week of being told their diagnosis to gather information on their experiences [38]. Interviews can take a number of forms, ranging from one-on-one semistructured interviews, to focus groups where data is collected from multiple participants at once. To identify key themes arising from them, interviews are typically analysed using thematic analysis.

Interviews allow more detailed exploration of themes compared with questionnaires: they are open-ended and can provide data on emergent themes that researchers had not considered prior to interview. It can be difficult to draw generalised conclusions, and the interviewer may unintentionally influence the responses elicited. In focus groups, group dynamics can interfere with that data collection [39]. Analysis of qualitative interview data can be challenging; there is wide variation in the methods used.

#### Vignette studies

Vignette studies permit study of how patients respond to well-controlled manipulations in communication variables. These studies use ‘analogue patients’ (APs) who watch or read vignettes and imagine themselves to be in the position of the patient in the scenario. These APs can be healthy people, or current/previous patients [40]. APs observe simulated or real patient/doctor interactions (or read about them in written vignettes); communication variables are manipulated between different vignettes. APs then complete questionnaires or are interviewed to assess outcome measures. Several stages are involved: developing a valid script, designing valid manipulations, converting the scripted consultation to video, and administering the videos [7, 40]. 

A simple example of such a study is demonstrated by Bhise *et al*: participants were asked to read three separate vignettes, which had been developed to vary in the way physicians discussed diagnostic uncertainty; participants were randomly assigned to one of the three vignettes and then answered a questionnaire assessing perceived technical competence of physician, trust and confidence, visit satisfaction and adherence to physician instructions [24]. 

Vignette studies often attempt to manipulate one specific aspect of communication, while holding all other elements constant, to draw conclusions about cause and effect [7]. This allows standardisation and a greater degree of control over experimental manipulations compared with real consultations [8]. It can, however, oversimplify the complexity of actual doctor–patient communication: ‘isolating specific communication elements means employing a reductive approach, which may oversimplify the rich and complex reality of communication’ [7]. 

Vignette studies provide a more ethically acceptable alternative to manipulating communication in real consultations, particularly when the studying potentially harmful communication behaviours: as Hillen *et al* note, ‘in a study manipulating physicians’ information giving behaviour, it may be expected that a lower amount of information could negatively impact on real patients, making a lab setting more appropriate’ [7]. Ceiling effects (of patients being unwilling to criticise their own doctors) [41] can be overcome by using APs [8]. It is important to note that the long-term doctor–patient relationship may influence communication, which cannot be captured by AP studies [8]. 

Internal validity is determined by the extent to which manipulations are successful (ie, are the intended variations in communication style or content perceived as such?). It can be tested by asking participants to score the vignette on the manipulated variable using a variety of adjectives, or by using a validated scale [7, 8]. External validity—whether the findings are generalisable—depends on whether the reactions of APs are equal to clinical patients’ reactions [8]. This can hinge on the extent to which APs are able to adopt the vignette-patient’s perspective; whether APs can adequately imagine themselves having certain chronic conditions has been questioned [42]. Written vignettes have been criticised for their relatively low external validity, as they are less immersive than video vignette studies and can result in oversimplification of complex information [43]. To increase external validity, vignette characters can be matched to the participant group: background characteristics influencing preferences—for example, gender, education—must be taken into account [8, 43]. External validity can be measured by asking participants to answer questions such as ‘I think these films are realistic’ [7]. 

Overall, the potential limitations of vignette studies—and how they might be mitigated—must be considered. Vignette studies can incur significant costs (particularly when actors are used). A review of video vignettes concluded that ‘no gold standard exists for how best to tackle many of the methodological issues. Many of the published studies failed to… justify a number of their choices. There is little empirical evidence for the exact effects of the various possible approaches.’ [7] 

#### Observation and analysis of interactions

Observation and analysis of doctor–patient interactions provides a method of directly collecting data on communication. As with questionnaires, the validity of the specific instruments used to measure communication variables in observed interactions must be considered—the methodological quality of instruments used in the literature has been found to be variable [33]. 

#### Simulated patient studies

These studies use real clinicians, with simulated or standardised patients (SP). They can provide information on how doctors communicate in response to patient cues or in specific clinical situations: they allow direct study of real healthcare professionals communicating in controlled environments. The use of SP scripts results in less variation compared with observational studies of real patients, and thus greater comparability between healthcare professionals [15, 44]. 

As with vignette studies, there can be concerns over internal and external validity: the manipulations in SP behaviours/cues need to be perceived as intended by the healthcare professional.

SPs are sometimes unannounced (they appear in the doctor’s clinic list mixed in with real patients) and the consultations are covertly recorded [45]. This can increase external validity by making the simulated consultation as realistic as possible: the doctors should treat the SPs as real patients. For example, in a study examining communication in response to patient worries, two unannounced SPs visited primary care physicians as part of their normal clinics, and the consultations were recorded with a hidden device [45]. Participating physicians were asked if they could identify the SPs, and asked to rate them for realism: 40% were able to identify the SPs, but rated them as “very realistic”.

However, SP scenarios tend to be initial visits, so may not be representative of how physicians communicate with established patients [15]. The authors of one study noted that while simulated patients ‘provided a realistic proxy for patients with a wide variety of specialty-specific conditions…, they did not provide the range of patient characteristics that are encountered across everyday clinical practice.’ [46] 

#### Observations of real consultations

These studies involve observing real patient/doctor interactions. They can reflect actual clinical practice to a greater extent than simulated patient or vignettes studies. Some techniques (such as conversational analysis) allow detailed examination of how communication occurs in specific settings in practice [47]. Moreover, there can be examination of communication in the context of real doctor–patient relationships: the interaction between the length of the relationship and communication can be studied, in contrast to experimental techniques such as vignette or SP studies.

The consultations themselves are observed or audio-video-taped, and communication behaviours are measured and analysed. As with simulated patient studies, there is wide variation in how communication behaviours in the observed consultation are measured and analysed: coding systems, interactional analyses, checklists and rating scales are four ways of handling data from recorded or directly observed clinical encounters [15]. 

These studies involve real doctors/patients who must give consent, which may create a bias (those who consent may systematically differ from those who do not) [15]. The Hawthorne effect must also be mitigated against where possible: both doctors and patients modify their behaviour when they know that they are being observed, and some behaviours may be more sensitive to these effects than others [15]. A ‘bedding-in period’ where consultations are observed or recorded, but the data is not used allows some acclimatisation for the healthcare professionals involved, so that they can become more comfortable with being observed. For patients, it is more challenging to mitigate the Hawthorne effect.

These studies generally do not allow for controlled manipulation of relevant variables, so are more commonly observational rather than interventional. They can provide information about correlations between doctor and patient communication behaviours and different outcomes measures; they do not provide any information about causation. There is a need to carefully control for confounding factors (such as patient’s baseline health) [21], as ‘apparent associations between patient participation in the clinical encounter and better health outcomes could occur if sicker patients participate less’ [26]. 

## Conclusion

By considering the scientific merits and limitations of different study designs, attempts can be made to mitigate the problems associated with them in order to produce higher quality research. Here, we have outlined an approach to the study of doctor–patient communication, thorough an overview of the available tools. Different study designs could be used in combination to facilitate a more nuanced understanding of doctor–patient communication as a whole.

The importance of communication is emphasised in medical education [48, 49] but research often focuses on evaluating the extent to which interventions—such as specific training programmes—have an impact on student/doctor communication behaviours [50–52]. The extent to which communication skills taught in training environments are transferred to clinical environments has been questioned [53, 54]; it is also still unknown whether this training has a real-world impact on patient-focused outcomes.

It is important for researchers to consider what specific aspects of communication they intend to focus on (content, style or outcomes), choosing a specific methodology—or a combination of methodologies—to assess this appropriately. In this way, future researchers will be able to use combinations of them more creatively to produce evidence which is richer, more robust, and more relevant both to training and to clinical practice.

List of learning points

- Doctor–patient communication is important, but is challenging to study, in part because it is multifaceted.
- Communication can be considered in terms of both the elements which make up the communication itself, and its measurable effects. These effects are themselves varied: they can be proximal or distal, and can focus on subjective measures (how patients feel about communication), or objective measures (exploring more concrete health outcomes or behaviours).
- There are a wide range of available methodologies for the study of doctor-patient communication. To study doctor–patient communication more effectively, two or more different study designs could be used in combination.
- Researchers should understand their respective logistical advantages/disadvantages and scientific merits/limitations: both to understand current research, and to conduct robust and relevant studies in the future.
